# Supplementary material for: Tumor Growth Ameliorates Cardiac Dysfunction
Source: Cells. 2023 Jul 14;12(14):1853. doi: 10.3390/cells12141853 (PMC10378697; doi:10.3390/cells12141853)
Supplement: Supplementary file 1 [file cells-12-01853-s001.zip › cells-2456873-supplementary.pdf]

Supplementary material

Supplemental Figures:

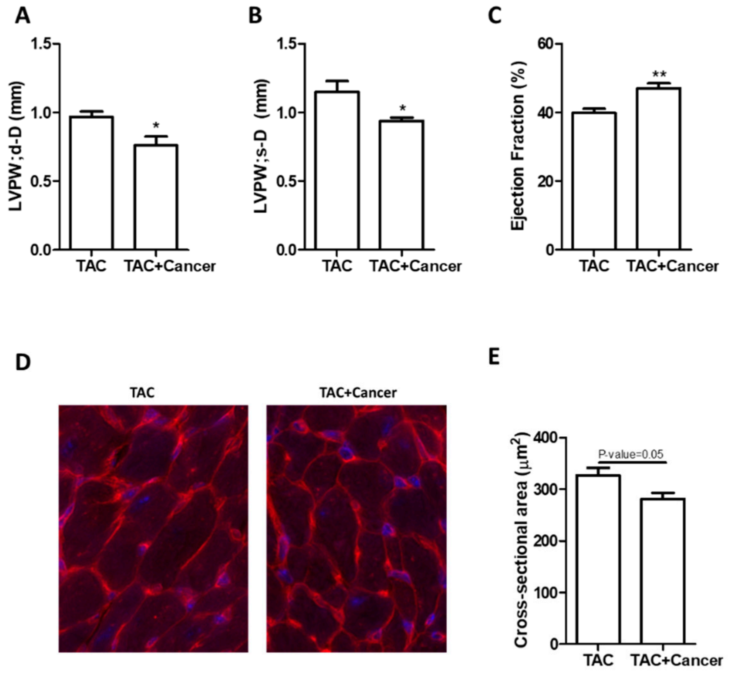

Supplemental Figure S1: Echocardiography parameters and cell size analysis.

A *Left ventricular posterior wall thickness in diastole (mm).* **B** *Left ventricular posterior wall thickness in systole (mm).* **C** *Left ventricular ejection fraction (%)* **D** *Female mice were either injected with PyMT cancer cells or left untreated. Five days after, all mice underwent TAC surgery for 3 weeks and hearts were analyzed. Ventricles sections were stained with FETC-labeled wheat germ agglutinin and cell size was analyzed. Scale bar=50µm.* **E.** *Quantification of cell size from D represented as cross sectional area in µm2. Results are presented as mean presented as mean ± SEM, student-t test. * P< 0.05, **P<0.01.*

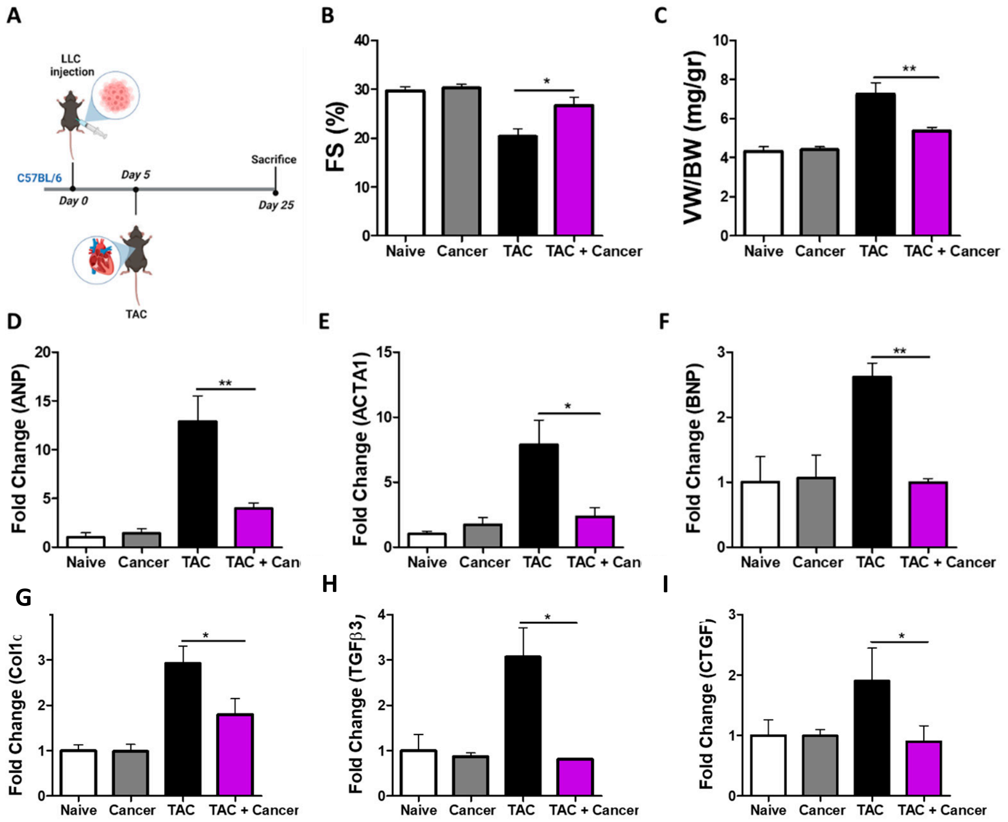

Supplemental Figure S2: LLC Tumor growth improves cardiac dysfunction following TAC in male mice.

A. *Schematic diagram of the experimental timeline. The 4 experimental groups included are naïve mice, LLC-injected mice, TAC-operated either LL-bearing or non-bearing mice.* ***B.*** *Fractional shortening (FS) of the experimental cohorts, calculated from echocardiography data, at day 25. Calculated based on parameters in Supp. Table 5.* ***C.*** *Ventricular weight/body weight ratio (VW/BW) of the experimental cohorts at day 25.* ***D-I.*** *The mRNA levels of hypertrophic markers ANP and BNP and ACTA1 (D-F) and fibrosis markers collagen, TGFβ3, and CTGF (G-I) in the hearts of the experimental cohorts at day 25, measured using qRT-PCR, normalized to the GAPDH housekeeping gene. The results are presented as mean ± SEM, student-t test. * P< 0.05, **P<0.01. (n=5 in all groups).*

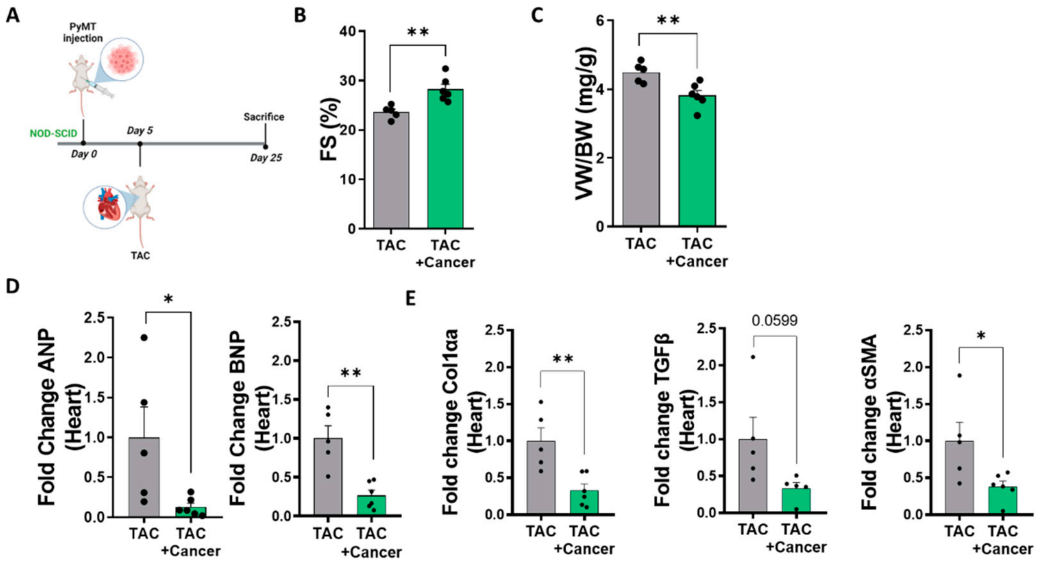

Supplemental Figure S3: Tumor growth's beneficial effect in NOD-SCID mice.
A. *Schematic diagram of the experimental timeline.* ***B.*** *Fractional shortening (FS) of TAC-operated PyMT-bearing and non-cancer bearing mice, calculated from echocardiography data. Calculated based on parameters in Supp. Table 6.* ***C.*** *The ventricular weight/body weight ratio (VW/BW) of TAC-operated PyMT-bearing and non-cancer bearing mice.* ***D-E.*** *The mRNA levels of hypertrophic markers ANP and BNP (D) and fibrosis markers collagen, TGFβ3 and αSMA (E) in the hearts of TAC-operated PyMT-bearing and non-cancer bearing mice, measured using qRT-PCR, normalized to the GAPDH housekeeping gene. The results are presented as mean ± SEM, student-t test. * P< 0.05, **P<0.01. In B-E, each dot represents one mouse. (n=5 in all groups).*

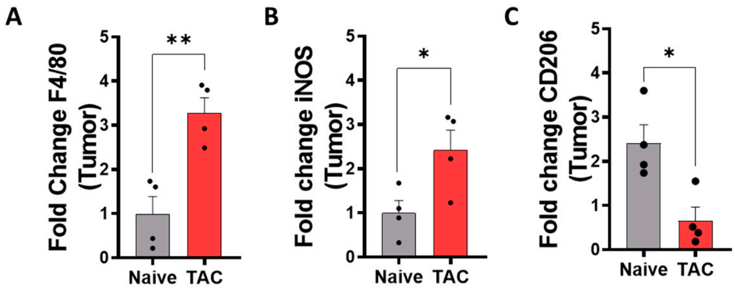

Supplemental Figure S4: M1 macrophage polarization in the tumor of TAC-operated mice.

A-C. *Tumor mRNA levels of macrophage markers F4/80 (A), iNOS (B), CD206 (C), in the PyMT tumor of TAC-operated (n=4) and non-TAC-operated (naïve) (n=4) mice, measured using qRT-PCR, normalized to the Hsp90 housekeeping gene. The results are presented as mean ± SEM, Student t-test. * P< 0.05, **P<0.0. Each dot represents one mouse.*

A

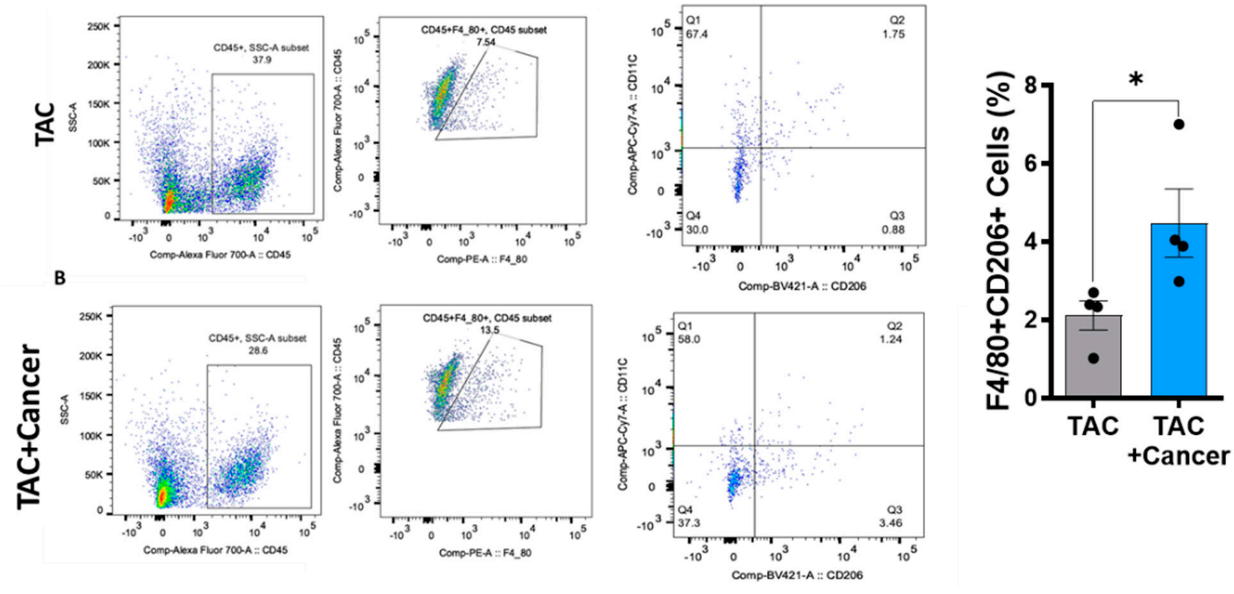

B

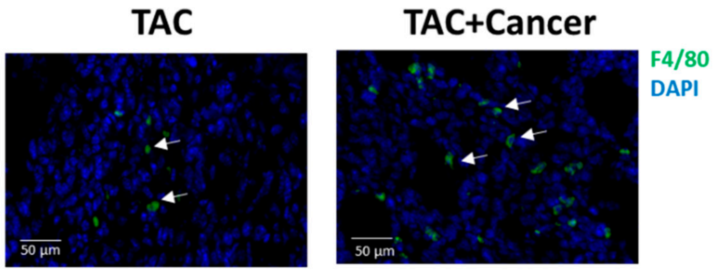

Supplemental Figure S5: Tumor growth induces cardiac macrophage polarization
A. *Gating strategy (right) and CD45+F4/80+CD206+ cell count (%) (left) in TAC-operated (n=4) and TAC-operated PyMT-bearing (n=4) mice hearts using flow cytometry. Each dot represents one mouse.* ***B.*** *Representative heart sections of a TAC-operated PyMT-bearing and non-cancer-bearing mouse hearts stained with F4/80, arrows indicate to positive cells. Arrows indicate for F4/80 positive cells. The results are presented as mean ± SEM, Student t-test. * P< 0.05.*

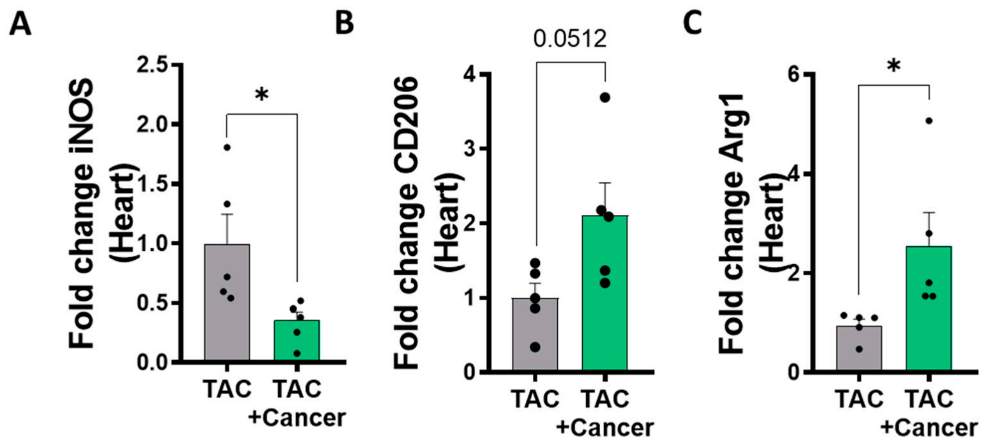

Supplemental Figure S6: M1-to-M2 polarization in NOD-SCID mice.
A-C. *mRNA levels of macrophages markers iNOS (A), CD206 (B) and ARG1 (C) in the hears of TAC-operated PyMT-bearing (n=5) and non-tumor-bearing (n=5) mice, measured using qRT-PCR, normalized to the GAPDH housekeeping gene. The results are presented as mean ± SEM, Student t-test. * P< 0.05. Each dot represents one mouse.*

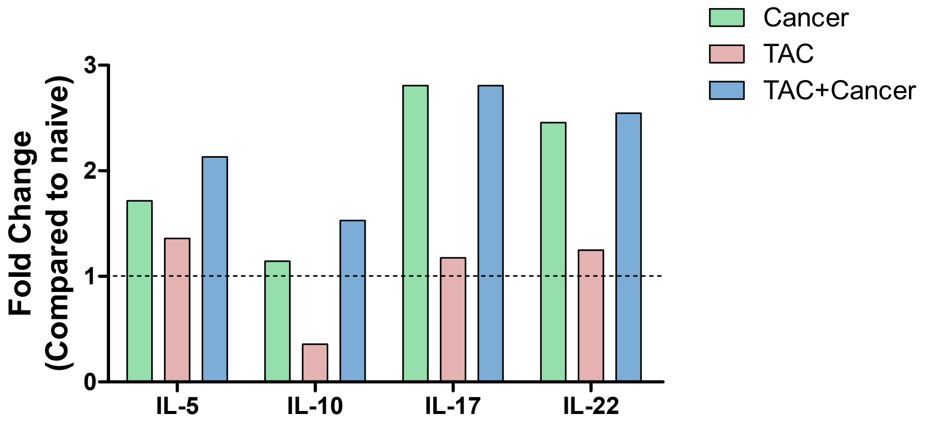

Supplemental Figure S7: M2 polarizing cytokines are elevated in the serum of TAC-operated PyMT-bearing mice.
Blood serum of 4 groups: naïve, PyMT bearing (Cancer), TAC-operated PyMT-bearing (TAC+cancer) and non PyMT-bearing (TAC) was used to probe cytokine array. For each cytokine, serum level is presented as fold change relative to naïve mice's blood serum. (n=4 in all groups). Dashed line indicates to 1 Fold change.

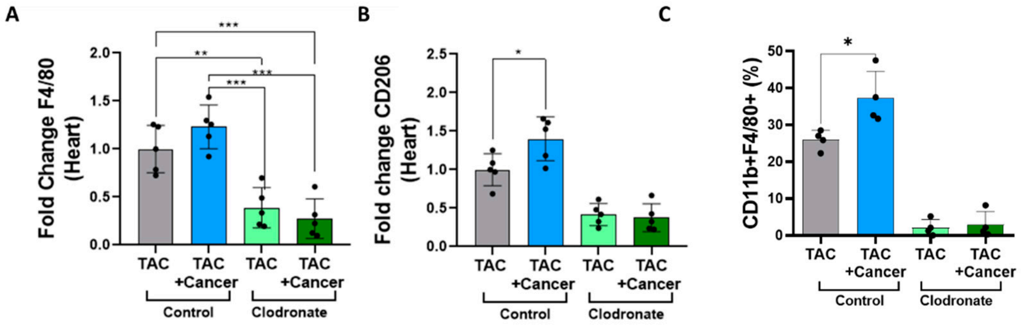

Supplemental Figure S8: Efficiency of macrophage depletion by clodronate liposomes.

A-B. *Expression levels of macrophage markers F4/80 (A) and CD206 (B) in the hearts of TAC-operated PyMT-bearing and non-tumor-bearing mice, measured using qRT-PCR, normalized to the HSP90 housekeeping gene.* ***C.*** *CD11b+F4/80+ cell count in the heart of TAC-operated PyMT-bearing and non-tumor-bearing, measured using flow cytometry. The results are presented as mean ± SEM, Student t-test. * P< 0.05, **P<0.01, ***P<0.001. Each dot represents one mouse. (n=5 in all groups).*

Gene	Forward	Reversed
Hsp90	TCGTCAGAGCTGATGATGAAGT	GCGTTTAACCCATCCAACTGAAT
GAPDH	TTGCCATCAACGACCCCTTCAT	AGACTCCACGACATACTCAGCA
ANP	GCTTCCAGGCCATATTGGAG	GGGGGCATGACCTCATCTT
BNP	GAGGTCACTCCTATCCTCTGG	GCCATTTCCTCCGACTTTTCTC
ACTA-1	GTGAGATTGTGCGCGACATC	GGCAACGGAAACGCTCATT
Col1α1	CTGGCGGTTCAGGTCCAAT	TTCCAGGCAATCCACGAGC
TGFβ3	CCTGGCCCTGCTGAACTTG	GACGTGGGTCATCACCGAT
CTgF	AGACCTGTGGGATGGGCAT	GCTTGGCGATTTTAGGTGTCC
αSMA	GTCCCAGACATCAGGGAGTAA	TCGGATACTTCAGCGTCAGGA
F4/80	CCCCAGTGTCCTTACAGAGTG	GTGCCCAGAGTGGATGTCT
iNOS	GACATTACGACCCCTCCCAC	GCACATGCAAGGAAGGGAAC
CD206	CTAACTGGGGTGCTGACGAG	GGCAGTTGAGGAGGTTCAGT
Arg1	AATGAAGAGCTGGCTGGTGT	CTGGTTGTCAGGGGAGTGTT
CD163	CCTCCTCATTGTCTTCCTCCTGTG	CATCCGCCTTTGAATCCATCTCTTG
INFγ	ACAGCAAGGCGAAAAAGGATG	TGGTGGACCACTCGGATGA
IL-13	AACGGCAGCATGGTATGGAGTG	TGGGTCCTGTAGATGGCATTGC
CCL2	GTGATGGAGGGGGTCAGGA	GGGATGGGACAGCCTAAACT
TNFα	CCCTCACACTCAGATCATCTTCT	GCTACGACGTGGGCTACAG

Supplemental Table S1: Oligonucleotides sequence

	TAC	TAC+Cancer
LVID;d-D	4.34 ± 0.28	4.12 ± 0.38
LVID;s-D	3.34 ± 0.26	2.96 ± 0.33
FS	23.03 ± 3.16	28.14 ± 2.6

Supplemental Table S2: Echocardiography parameters of Figure 1B

	TAC	TAC+TVI
LVID;d-D	3.79± 0.59	3.72 ± 0.01
LVID;s-D	2.96 ± 0.53	2.64 ± 0.08
FS	22.29 ± 1.75	28.98 ± 2.08

Supplemental Table S3: Echocardiography parameters of Figure 3D

	Control		Clodronate	
	TAC	TAC+Cancer	TAC	TAC+Cancer
**LVID;d-D**	4.044 ± 0.36	3.94 ±0.27	3.94 ±0.24	3.93 ±0.15
**LVID;s-D**	3.078 ± 0.27	2.8 ±0.19	3.02 ±0.25	2.96 ±0.12
**FS**	23.83 ± 2.63	28.81 ±1.72	23.35 ±3.06	24.55 ±2.62

Supplemental Table S4: Echocardiography parameters of Figure 5C

	TAC	TAC+Cancer
LVID;d-D	4.29 ± 0.29	4.14 ± 0.24
LVID;s-D	3.42 ± 0.3	3.04 ± 0.21
FS	20.36 ± 2.9	26.73 ± 2.74

Supplemental Table S5: Echocardiography parameters of Supp. Figure 1B

	TAC	TAC+Cancer
LVID;d-D	3.47 ± 0.33	3.25 ± 0.2
LVID;s-D	2.65 ± 0.28	2.32 ± 0.18
FS	23.83 ± 1.22	28.77 ± 2.15

Supplemental Table S6: Echocardiography parameters of Supp. Figure 2B
